# Precision medicine and the cursed dimensions

**DOI:** 10.1038/s41746-019-0081-5

**Published:** 2019-02-04

**Authors:** Dennis L. Barbour

**Affiliations:** Departments of Biomedical Engineering, Computer Science and Engineering, Neuroscience and Otolaryngology, Washington University in St. Louis, St. Louis, MO 63130 USA

**Keywords:** Translational research, Diagnostic markers

## Abstract

Our intuition regarding “average” is rooted in one-dimensional thinking, such as the distribution of height across a population. This intuition breaks down in higher dimensions when multiple measurements are combined: fewer individuals are close to average for many measurements simultaneously than for any single measurement alone. This phenomenon is known as the curse of dimensionality. In medicine, diagnostic sophistication generally increases through the addition of more predictive factors. Disease classes themselves become more dissimilar as a result, increasing the difficulty of incorporating (i.e., averaging) multiple patients into a single class for guiding treatment of new patients. Failure to consider the curse of dimensionality will ultimately lead to inherent limits on the degree to which precision medicine can extend the advances of evidence-based medicine for selecting suitable treatments. One strategy to compensate for the curse of dimensionality involves incorporating predictive observation models into the patient workup.

Initially, all airplane cockpits were designed the same, sized to fit the “average pilot.” Investigating high crash rates in fighter jets, the newly formed US Air Force eventually realized that 0 of its 4000 jet pilots fell within 15% of average on 10 relevant body measurements (i.e., 10 dimensions), and fewer than 150 fell within 15% of average on the three most important measurements.^1^ Even for this relatively homogeneous group of young men, the concept of average body shape was unhelpful for designing effective cockpits because no one is average across many dimensions. Designing for the average was literally designing for no one. The solution for this problem is as obvious today as it was radical then: outfit cockpits with adjustable seats, thereby individualizing seating position for each pilot. Once the pilots could fully reach their flight controls during strenuous aerial maneuvers, the mysterious crashes ended.

Modern medical approaches seek to personalize diagnoses and therapies for each patient, but current strategies to do so share much in common with designing cockpits for average body shapes. Broadly speaking, evidence-based medicine relies on empirical findings from scientifically designed studies measuring one or a few predictive factors and evaluating mainly group average outcomes.^2^ Precision medicine adds correlation studies using large databases and many factors, but still typically exploits group average inference to draw conclusions, albeit with smaller, more cohesive groups.^3^ Strategies to personalize medical workups by averaging the most similar patients can be effective but suffer the curse of dimensionality.

Using more factors to stratify patients into finer subdivisions means that fewer previous patients will fall into any given high-dimensional category for useful comparison. For example, a patient with 10 independent risk factors each with 10% prevalence implies that the probability of finding a similar previous patient to compare against is 1 in 10 billion. No matter how big our databases of patient records become, we will eventually exhaust our ability to make better medical decisions for future patients by simply evaluating ever more factors in previous patients to find similar antecedents.

Consider that the relationship between smoking and health is one of the largest effect sizes in medicine.^4^ Even with such a profound impact on the cardiopulmonary system, however, not everyone dies prematurely from smoking.^5^ Although impactful as a public health initiative on the basis of the effect size, advice to cease smoking may therefore not be universally valuable advice for every single patient. This disparity between average and individual is even more prominent for weaker effects where interpatient variability is likely to be higher.

A reasonable approach to help personalize medical advice about smoking might be to assess the commonalities that smoking pensioners share, thereby identifying putative predictive factors about outcomes from smoking. The curse of dimensionality pitfall here is that in the nonintuitive high-dimensional combinatorics of genetics, environment, and lifestyle, it is possible that no two smoking pensioners are truly similar. In other words, perhaps many combinations of factors can lead to healthy outcomes in smokers, and perhaps every smoking pensioner has a different combination. Seeking commonalities, while logical in lower dimensions, may in higher dimensions primarily reveal random effects that do not replicate for other similar cohorts. In the specific case of smoking and cancer, evidence of discernible high-dimensional interactions among predictive factors does exist.^6^ In other scenarios with weaker effects or smaller clinical populations, however, the limitations of cohort-level analysis methods may simply prevent determination of clinically meaningful distinctions.

Current approaches to improve medical inference operate within the evidence-based medicine framework to select the most appropriate predictive factors^7^ or model structure^8^ given population data. These approaches lead to new understanding of relevant factors and improve average predictive accuracy, but are still limited by the curse of dimensionality in that they require larger populations to improve further.

Individualized assessments represent an alternative approach analogous to adjustable seating. My desk chair has 10 degrees of freedom, and when it is adjusted properly, it fits my body comfortably. Both theoretical constraints (e.g., the laws of physics) and empirical constraints (e.g., the ranges of human body sizes) govern the chair adjustments. Within those constraints I can construct an individualized model of my body shape and encode it with the appropriate levers and knobs. The chair configuration that best fits me has become an observation model for my body, predicting how comfortable or usable other configurations are likely to be for me.

Clinical observation models can combine theoretical knowledge, empirical findings from previous patients, and past observations from the current patient to deliver predictions regarding new observations from the current patient.^9^ They capture the multidimensional interactions of latent clinical factors specific to each patient.

Some clinical domains already make limited use of partial observation models, notably concerning disorders of hearing or vision.^10^ Take, for example, refractive errors requiring spectacle correction. The observation model in this case encodes the geometry of an individual eye’s light-bending apparatus. This model does not require information from any other patient’s eyes to achieve predictive accuracy, though such information could be useful in some contexts. Because the model also represents proper corrective lens parameters, it is referred to as a patient’s “prescription” for eyeglasses. This functional equivalence between observations and treatment obscures the general value that observation models can contribute toward individualizing medical care generally.

More complex clinical scenarios such as dementia, autism or cancer may present situations whereby treatment selection does not follow directly from an observation model. In those cases diagnosis models and treatment outcome models are added to yield a comprehensive clinical decision support framework referred to as Advanced Inferential Medicine^℠^ (AIM).^11^ Clinical decisions in this framework are not based on ever-larger databases of observations themselves, but “modelbases” instead. The curse of dimensionality still applies but is at least partially mitigated by using additional observations from patients to refine models of their physiology rather than attempting to place them within an exploding number of diagnostic categories, each defined by fewer previous patients.

Retaining established evidence-based medical inference principles in the precision medicine era will lead to steady improvements in patient outcomes. As databases come to encompass nearly the full complement of available patients, however, these improvements will slow and alternative forms of medical inference will become more valuable. Recognizing this trend now will enable data collected during this time period to be structured in a way that anticipates more individualized medical inference methods such as AIM in the near future.

## References

[1] Rose, T. *The End of Average: How We Succeed in a World That Values Sameness* (HarperCollins Publishers, New York, NY, 2016).

[2] Eddy DM (2005). Evidence-based medicine: a unified approach. Health Aff. (Millwood).

[3] National Research Council (US) Committee on A Framework for Developing a New Taxonomy of Disease. *Toward Precision Medicine: Building a Knowledge Network for Biomedical Research and a New Taxonomy of Disease* (The National Academies Press, Washington, DC, 2011). 22536618

[4] Pesch B (2012). Cigarette smoking and lung cancer—relative risk estimates for the major histological types from a pooled analysis of case-control studies. Int. J. Cancer.

[5] Cohen-Mansfield J (2013). Smoking and mortality among persons aged 75–94. Prev. Med..

[6] Levine ME, Crimmins EM (2016). A genetic network associated with stress resistance, longevity, and cancer in humans. J. Gerontol. A Biol. Sci. Med. Sci..

[7] Schneeweiss S (2009). High-dimensional propensity score adjustment in studies of treatment effects using health care claims data. Epidemiology.

[8] Rolling CA, Yang Y (2014). Model selection for estimating treatment effects. J. R. Stat. Soc..

[9] Barbour DL (2018). Formal idiographic inference in medicine. JAMA Otolaryngol. Head Neck Surg..

[10] Song XD (2015). Fast, continuous audiogram estimation using machine learning. Ear Hear.

[11] Barbour, D. L. Advanced Inferential Medicine℠. Open Science Framework. https://osf.io/4nmkv (2017).

